# Prophylaxis of Crohn's disease recurrence: A surgeon’s perspective

**DOI:** 10.1002/ags3.12368

**Published:** 2020-06-24

**Authors:** Dallas D. Wolford, Alessandro Fichera

**Affiliations:** ^1^ Division of Colorectal Surgery Department of Surgery Baylor University Medical Center Dallas Texas USA

**Keywords:** anastomosis, Crohn's disease, medical therapy, prophylaxis, recurrence

## Abstract

Management of inflammatory bowel disease has evolved extensively in the last three decades. We have learnt a lot about the pathophysiology and natural history of the disease. New effective classes of drugs with the associated potential morbidity have been introduced. New surgical techniques have been popularized leading to a better understanding of the optimal timing of surgery. The result is a very complex subspecialty of gastroenterology and colorectal surgery called the "IBDologist." Only if we manage these complex patients in the context of a multi‐disciplinary team will we be able to obtain outstanding outcomes, specifically with high and sustained remission rates for these patients.

## Graphical abstract

Management of inflammatory bowel disease has evolved extensively in the last three decades. We have learnt a lot about the pathophysiology and natural history of the disease. New effective classes of drugs with the associated potential morbidity have been introduced. New surgical techniques have been popularized leading to a better understanding of the optimal timing of surgery. The result is a very complex subspecialty of gastroenterology and colorectal surgery called the “IBDologist.” Only if we manage these complex patients in the context of a multi‐disciplinary team we will be able to obtain outstanding outcomes, specifically with high and sustained remission rates for these patients.

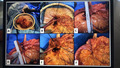

## 1 INTRODUCTION

Crohn's disease (CD) is a chronic, multifocal transmural inflammatory disease that affects any portion of the gastrointestinal tract. Clinically, it presents with intermittent abdominal pain, diarrhea, hematochezia, failure to thrive, fever and weight loss.^1^, ^2^ The pathophysiology of the disease is dictated by the interaction of environmental and personal factors including diet with effects on the microbiome and genetic predisposition. The natural history is multifaceted as the disease is associated with a spectrum of clinical phenotypes and complications requiring surgical intervention.^3^

## 2 PREVALENCE/INCIDENCE

Crohn's disease was initially thought to be a disease affecting primarily Ashkenazi Jewish patients, Northern European countries and the United States. It has been estimated that prevalence of CD in the United States is approximately 201/100 000 with an incidence of 3.1‐20.2/100 000.^4^ Currently, over 1.5 million patients in the United States and >2 million in Europe are known to be affected by the disease.^4^, ^5^, ^6^, ^7^ Over the past decade, the incidence of CD has been increasing particularly in populations previously regarded to be low‐risk, especially in Asia.^8^, ^9^

## 3 RECURRENCE CHARACTERISTICS AND RATES

In patients with Crohn's, the lifetime risk of surgery still approaches 80% despite the increasing use of anti‐TNF and immunosuppressive therapies.^10^, ^11^, ^12^ Intestinal resection is not curative, and there is a substantial risk of postoperative recurrence (POR). Nearly 25% of patients will require intestinal resection within 1 year from the time of diagnosis.^13^ Fifty percent of patients will have their initial surgical resection within 10 years of diagnosis, and 30% will require a second surgery.^14^. D'Haens et al^15^ demonstrated that microscopic recurrence has been observed as early as 1 week postoperatively. Seventy‐five to ninety percent of patients with CD will have endoscopic recurrence within 1 year of initial surgery, a majority of which will progress to have recurrence of symptoms clinically and require reoperation.^16^, ^17^, ^18^ Of the patients that have undergone a primary excisional surgery, 25% will require a subsequent procedure within 5 years, 35%–60% in 10 years.^12^, ^14^, ^19^ Historically, in the mid‐20th century, the overall rate of recurrence was 30%–34% with a higher risk associated with involvement of small bowel and early age of onset.^20^ Recurrence most often occurs just proximal to or at the anastomotic site and is categorized as subclinical or endoscopic, clinical, and lastly surgical.^17^, ^18^, ^21^, ^22^, ^23^ Endoscopic recurrence is graded by the Rutgeerts score^3^, ^18^, ^23^ based on evaluation of the neo‐terminal ileal intestinal mucosa after surgical resection for CD.^18^, ^19^ Normal ileal mucosa is designated i0; i1 for <5 aphthous ulcers; i2 for >5 aphthous ulcers with presence of normal intervening mucosa; i3 for presence of ulceration with grossly normal intervening mucosa; i4 for severe ulcerations, presence of nodules, cobblestoning, or strictures.^18^, ^23^ Endoscopic evidence of subclinical recurrence is typically detected early with the severity indicative of likely progression to clinical recurrence^23^ and it is detectable as early as 1 week after surgery.^15^, ^18^, ^23^ Clinical recurrence is defined as return of symptoms and is seen in 30% of patients within the first year of initial surgery, 5%–10% requiring reoperation less than a year after primary resection. Finally, surgical recurrence is the subsequent need for reoperation after initial bowel resection.

## 4 RISK FACTORS FOR RECURRENCE

Several factors have been described in the literature including active smoking, penetrating or perforating behavior, perianal disease, prior intestinal resection, extensive small bowel resection (>50 cm), type of anastomosis, postoperative complications, resection margins, age at diagnosis of disease, gender, location and duration of disease, granuloma, family history, CRP level, myenteric plexitis, NOD2/CARD15 mutation, increased TGF‐b, and low II10 mRNA level.^11^, ^12^, ^24^ Of these, there are five established predictors of post‐operative recurrence: penetrating phenotype, perianal disease, prior intestinal resection, extensive small bowel resection, and active smoking.^11^, ^25^

### 4.1 Smoking

Smoking is the only modifiable proven risk factor associated with more severe complications of penetrating and fibrostenotic CD, increased risk of surgical recurrence.^26^ A 6‐year study conducted by Cottone et al^27^ found that smoking was associated with higher clinical CDAI scores, endoscopic recurrence, and the only significant predictor of surgical recurrence. A meta‐analysis conducted by Kuenzig et al,^28^ showed a significant association between current smokers (CS) and need for first surgery (HR 1.27) when compared to those who had never smoked (NS). Furthermore, Reese et al pooled data from 30 studies from 1966 to 2007 and found CS to have an increased risk of clinical recurrence following initial resection (58.3% vs 39% *P* < .005) and an increased risk of reoperation within 10 years of the initial resection (55.5% vs 32.1% *P* < .001) than NS. Recurrence is more likely to be at the prior surgical site in CS than NS.^29^ In a cohort of 174 patients, Sutherland et al^30^ found 5‐ and 10‐year surgical recurrence rates in smokers to be 36% and 70% respectively when compared to NS (20%, 40%). In a retrospective study of 141 patients with ileocecal disease who underwent ileocolonic resection, NS had a recurrence‐free rate of 81% and 64% at 5 and 10 years, respectively, vs 65% and 45% in CS (*P* = .007).^31^ The authors also found the recurrence rate to be higher in heavy smokers, further support the role of active smoking.^31^ Kane et al^32^ looked at 59 patients undergoing primary resection and followed them for 250 weeks and found a 69% risk of clinical relapse at an average of 130 weeks postoperatively in CS vs 23% and 234 weeks in non‐smokers (OR 2.96). Additionally, Unkart et al^33^ found that patients actively smoking at the time of the initial operation had a 2.1 increased risk of relapse requiring reoperation. Smoking cessation at time of diagnosis may decrease risk of recurrence as evidence shows former smokers (FS) to have the same risk as NS.^28^, ^34^, ^35^ When comparing CS and FS, CS again had higher risk of clinical relapse (65.7% vs 42.4% *P* = .03) and reoperation within 10 years of initial surgery (26.8% vs 17.5%, *P* = .04). There were no statistically significant findings between FS and NS.^28^, ^29^

### 4.2 Perforating disease

Perforating phenotype is more aggressive, associated with more complications, and is described as acute/subacute with abscesses, or chronic with fistulae.^31^, ^36^, ^37^ Perforating disease is one of the most common indications for surgery and has the highest rate of surgical recurrence.^1^, ^33^, ^38^ In 2008, Simillis conducted a meta‐analysis of 13 studies comparing surgical recurrence and the need for reoperation between perforating and non‐perforating CD patients who have undergone a primary resection. There was a significantly higher rate of surgical recurrence in perforating phenotype (*P* = .002) with phenotypic concordance between the primary and recurrent presentation and shorter surgical recurrence‐free survival (1.7 vs 13 years).^38^ A meta‐analysis looking at postoperative medical management vs placebo in maintaining clinical remission showed that perforating disease had greater endoscopic recurrence scores in the placebo group (*P* < .05).^39^

### 4.3 Small bowel disease

Ileocolonic disease is the most common presentation and thus most common site of surgical resection.^21^ Bernell et al^5^, ^40^ reported increased RR of POR to 1.8 and 1.5 (95% CI: 1.1‐2.0) respectively in patients with small bowel or ileocecal disease compared to colonic. Manser et al^41^ found ileal disease to be a predictive factor for reoperation (OR 2.42, *P* = .05). Ileal disease was associated with higher POR in a study by Borley et al^42^, and finally, a Swedish study evaluated the cumulative POR rate for ileal, ileocolonic, and colonic CD at 5, 10, and 15 years and found ileal and ileocolonic disease to have significantly higher POR than colonic disease.^43^ Ileocolonic CD confers a shorter interval between onset of symptoms and initial operation associated with higher POR.^44^

### 4.4 Perianal disease

The documented prevalence of perianal/anorectal involvement varies from 10% to 40%.^45^, ^46^ Isolated perianal disease is rare and is present in <5% of patients^47^ and conversely 15% of patients with ileocolonic disease and over 90% with colonic disease will have concomitant perianal involvement.^46^ Presence of perianal disease typically confers a more debilitating course with more frequent and often more severe extraintestinal manifestations and tends to be less responsive to conventional steroid therapy.^24^, ^48^, ^49^ Treatment involves both medical and surgical modalities; antibiotic bridge to immunosuppressive therapy and exam under anesthesia with abscess drainage and commonly, seton placement.^50^ The success of surgical treatment is dependent upon the presence of active proctitis. In a recent study, patients with rectal disease had a significantly higher rate of proctectomy than patients with rectal sparing (77.6% vs 13.6%, *P *< .0001).^51^

### 4.5 Duration of disease

Patients are stratified based on the risk of POR in three categories: low‐risk patients >10 years of disease, <10 cm segment of stricture, and no history of prior surgery or first surgery; moderate‐risk <10 years since diagnosis, >10 cm segment of intestinal stricture or narrowing, non‐perforating disease, no history of multiple surgeries; high‐risk patients are smokers, have perforating disease and history of multiple surgeries.^52^ Similarly, work by Yamamoto^53^ supported the theory that a shorter disease course from the time of diagnosis to initial surgery was a strong predictor of POR. However, what constituted a “short” duration of disease has yet to be agreed upon, but is generally thought to be <10 years. Poggioli^54^ found that preoperative disease duration of <6 years was associated with higher surgical recurrence rates postoperatively. Another study by Chardavoyne et al^55^ found higher recurrence rates between 3 and 10 years of disease duration. In contrast, a pediatric study found that patients who underwent primary resection within 1 year of symptom onset experienced delayed recurrence (30% in 8 years), compared to those who underwent surgery between 1 and 4 years after symptom onset (50% in 4 years) and those who had surgery 4 or more years after symptom onset (50% in 3 years).^56^ While it remains unclear whether disease duration plays a role in surgical recurrence, a shorter duration between onset of symptoms and resection indicates a likely more aggressive phenotype.^53^

### 4.6 Age at diagnosis

The association of age at diagnosis and POR has been frequently reported in the literature. The younger a patient the more aggressive and disabling the disease course.^57^ Scarpa et al^58^ conducted a retrospective review of 120 patients and found younger age at diagnosis was a risk factor for POR. Dombal found the highest recurrence rate in younger children and adolescents, lower in adults, and lowest rates of recurrence in patients >60 years.^19^

### 4.7 Microbiome

The investigations of role of the fecal microbiome in POR is at its infancy. It is believed that the fecal and intestinal microbiota is an antigenic driver of CD recurrence.^59^ Hamilton et al^59^ reported a significant increase in the diversity of microbiota after resection and an association with disease remission. Specifically, a decrease in Clostridiales and Lactobacillales was associated with a negative association with POR and a decrease in Actinobacteria, Bacteroides, and Bacteroidetes showed a positive association with POR.^59^ Wright et al^60^ showed that endoscopic recurrence at 6 months was associated with increased abundance of Proteus species when compared to those in remission (*P* = .008) and a decrease of Faecalibacterium to be a risk factor for endoscopic recurrence at 18 months (*P* = .013). It is clear that there are profound changes in the intestinal microbial community following resection for CD, what is not yet clear is which specific alterations lead to dysbiosis and dysfunction of mucosal barrier and how this can be implemented clinically.^59^, ^60^

### 4.8 Nutritional supplementation with elemental diet

Enteral nutrition with an elemental diet is widely utilized in Japan preoperatively for disease control and maintenance of remission^61^, ^62^ and nutritional optimization, even though a recent meta‐analysis suggested it to be inferior to corticosteroid treatment.^63^ In surgically induced remission enteral nutrition has been shown to prevent POR.^64^, ^65^ With a better understanding of the role of the microbiome, it would be interesting to investigate the impact of elemental diet on the gut microbiome to elucidate potential mechanisms of recurrence prevention.

## 5 SURGICAL PROPHYLAXIS

In a case‐controlled comparative analysis, 138 patients were divided between wide‐lumen stapled side‐to‐side and hand‐sewn end‐to‐end anastomoses.^66^ Clinical recurrence was noted in 24% of side‐to‐side anastomoses and 57% of end‐to‐end anastomoses with a cumulative surgical recurrence rate of 11% and 20% at 5 years (*P* = .017). The authors concluded that the side‐to‐side anastomosis created a wider diameter lumen that reduced clinical recurrence by limiting fecal stasis and reducing recurrence secondary to ischemia. A subsequent meta‐analysis of eight studies, with 661 patients, and 712 anastomosis compared multiple types of anastomoses. Side‐to‐side anastomosis had fewer leaks and reduced overall postoperative complications and length of hospital stay. The overall and surgical recurrence rates of these groups, however, were similar.^67^ Several retrospective studies were subsequently performed and confirmed no difference in anastomotic leak rates or surgical recurrence between the two groups^68^, ^69^ or, as in the study by Feng et al,^70^ demonstrated significant decrease in surgical recurrence in the side‐to‐side anastomosis group. The CAST trial published in 2009 was the first randomized controlled trial comparing outcomes from side‐to‐side vs end‐to‐end anastomoses and found an endoscopic recurrence rate of 37.9% and 42.5% (*P* = .55), symptomatic recurrence rate of 22.7% and 21.9%, respectively (*P* = .92).^71^ This trial was underpowered to show a difference.

In an attempt to decrease the rate of surgical recurrence, Kono et al^72^ developed a new anastomotic technique that preserved a greater portion of the mesenteric neurovasculature and implemented a novel supporting column posterior to the anastomosis. Rather than removing a large segment of mesentery down to the mesenteric root, Kono used a tissue sealer to create a small window just inferior to the border of the intestinal wall along the length of the bowel to be excised. This method kept a greater portion of the vasculature and innervation intact allowing for improved healing and function. Furthermore, the antimesenteric anastomosis results in a wide lumen, irrespective of the intestinal caliber of the two segments. The first publication by Kono included 69 patients who underwent the Kono‐S and compared them with conventional anastomoses. No patients in the Kono‐S group had surgical recurrence at 5 years vs 15% in the conventional group (*P* < .0013).^72^ Two large multicenter studies reported only two surgical recurrences at 65 months and a 98.6% recurrence‐free survival for 5 and 10 years in the Kono‐S group.^73^, ^74^ Recently, the first randomized controlled trial to compare Kono‐S and side‐to‐side anastomoses confirmed a reduction in the endoscopic and clinical recurrence in favor of the Kono‐S.^75^ Additionally, the Kono‐S patients who developed endoscopic recurrence had significantly lower Rutgeerts scores (*P* = .03).^75^ The opposite approach of a wide mesenteric resection was recently published in a small cohort study of 64 patients.^76^ The difference in POR was 2.9% vs 40% (*P* = .003) in favor of the wide mesenteric excision. While details of the type of anastomoses utilized are not available in the study, wide excision should be compared in a prospective and randomized fashion with the Kono‐S anastomosis.

## 6 MEDICAL PROPHYLAXIS

Medical therapy—immunosuppressive modulators and biologic agents—have been associated with decreased need for operation in the short‐term, without definitive long‐term evidence.^77^ In addition to antibiotics and immunomodulators, several studies have demonstrated anti‐tumor necrosis factor to be efficacious in reducing POR in CD,^52^, ^78^, ^79^, ^80^, ^81^, ^82^, ^83^, ^84^, ^85^ with a more pronounced effect noted in anti‐TNF naïve patients.^86^. In 2016, Regueiro et al^87^ published the PREVENT trial to evaluate the efficacy of infliximab in preventing POR after ileocolic resection. Two hundred and ninety‐seven patients from 2010‐2012 were included and evaluated for clinical and endoscopic recurrence at 76 weeks. The primary endpoint of clinical recurrence was not met, but 30.6% of the infliximab group was found to have endoscopic recurrence vs 60% in the placebo group (*P* < .001).^87^ Since early endoscopic recurrence correlates with surgical recurrence,^88^, ^89^ it may be extrapolated from this study that infliximab and adalimumab have the potential to reduce clinical POR.^90^, ^91^ The unsolved dilemma is who is going to benefit the most from an expensive and potentially morbid therapy. To address this question, De Cruz published the Post‐Operative Crohn's Disease Endoscopic Recurrence (POCER) trial to compare early endoscopic surveillance in patients and escalation of medical therapy based on preoperative risk stratification with standard conservative management.^92^ Patients were categorized as high‐ or low‐risk then randomized for 6‐ vs 18‐month initial colonoscopy following surgery. High‐risk patients were treated with either thiopurine or and all patients were treated with 3 months of metronidazole. If endoscopic recurrence was observed in any of the patients in the 6‐month group, they received additional treatment. At 18 months, the patients who underwent initial ileocolonoscopy at 6 months and additional treatment if indicated had an endoscopic recurrence rate of 49% vs 67%, an 18% reduction. Endoscopic recurrence in the high‐risk group at 6 months was 45% for those receiving thiopurine and 21% with adalimumab. Two conclusions can be drawn from these findings: patients considered to be high‐risk benefit from shorter intervals of postoperative surveillance; if postoperative medical prophylaxis is indicated, anti‐TNF therapy should be considered in high‐risk patients.^87^, ^92^, ^93^, ^94^

## 7 DISCUSSION

Surgical resection in CD is not curative and recurrence is common; thus, maximizing preventative efforts to reduce POR is essential. Recurrence occurs endoscopically first, followed by return of symptoms resulting in a second operation. Identifying high‐risk patients is critical to initiate or resume medical therapy in a timely manner. It is important to look at the disease characteristics that are proven risk factors for POR and intervene on the modifiable factors like smoking. Surgery has to be performed following the principles of wide lumen anastomosis and avoidance of postoperative complications. The initial step in postoperative CD management is risk stratification. Early postoperative endoscopy for high‐risk patients with intensification of medical therapy is associated with 18% lower rate of endoscopic recurrence. By targeting high‐risk patients with early colonoscopy, unnecessary and costly treatments can be avoided in low‐risk patients. While there is no concrete evidence that smoking decreased therapeutic response to biologic therapy, there is a two times increased risk of POR recurrence in active smokers either incapable or unwilling to stop.^95^

## DISCLOSURE

Conflict of Interest: The authors declare no conflict of interests related to this article.
